# MATURE CYSTIC TERATOMA OF THE PANCREAS: AN UNUSUAL INDICATION FOR LAPAROSCOPIC DISTAL PANCREATECTOMY

**DOI:** 10.1590/0102-672020220002e1693

**Published:** 2022-11-14

**Authors:** César Munoz, Cristian Lindner, Felipe Pizarro, Carlos Pino

**Affiliations:** 1 Regional Hospital, Department of Hepatobiliary and Pancreatic Surgery – Talca, Chile; 2 Catholic University of Maule, Medicine Faculty – Talca, Chile; 3 Regional Hospital, Radiology Department – Talca, Chile; 4 Regional Hospital, Pathology Department, Talca, Chile.

**Keywords:** Pancreatectomy, Teratoma, Dermoid Cyst, Pancreatic Neoplasms, Pancreatectomia, Teratoma, Cisto Dermoide, Neoplasias Pancreáticas

## INTRODUCTION

Mature cystic teratomas are benign and congenital slow-growing tumors of germ cell origin that may arise from any of the germinal layers encapsulated in a well-defined cystic wall^15,16^. As most mature cystic teratomas are composed mainly of ectodermal components, they are also referred to as dermoid cysts^15^. At present, its clinical course is known to be strictly benign due to the well-differentiated parenchymal tissues lined on its cystic wall, which drives its benign biological behavior^9^. However, despite its benign nature, the complete surgical excision is currently considered the standard of care^6,9,16^.

The objective of this study was to report a case of a 72-year-old male with incidentally detected cystic mass arising from pancreas tail, who underwent minimally invasive distal pancreatectomy for its resection.

## CASE REPORT

**Figure 1:**
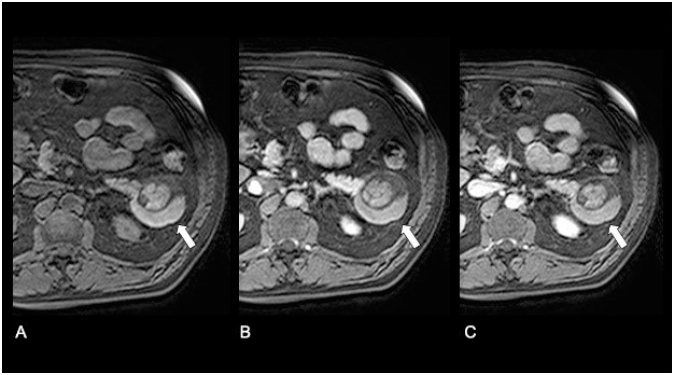
Axial T1 - weighted images. (A) Noncontrast. (B) Arterial phase. (C) Portal venous phase. Mild peripheral enhancement with heterogeneous internal content in the absence of malignant suspected nodular areas (arrows).

**Figure 2:**
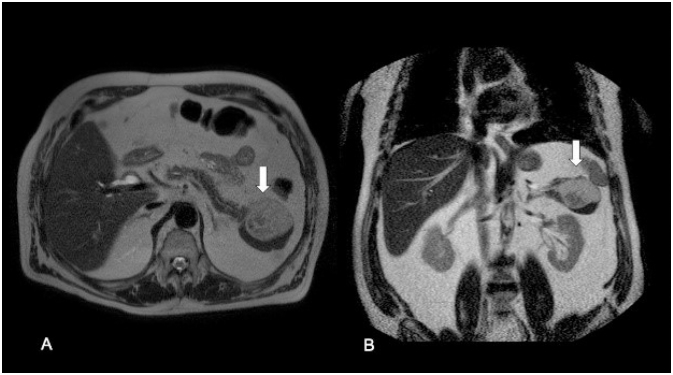
T2-weighted images. (A) Axial. (B) Coronal. Heterogeneous and well-defined solid mass in pancreatic tail (white arrows). No ductal.

**Figure 3:**
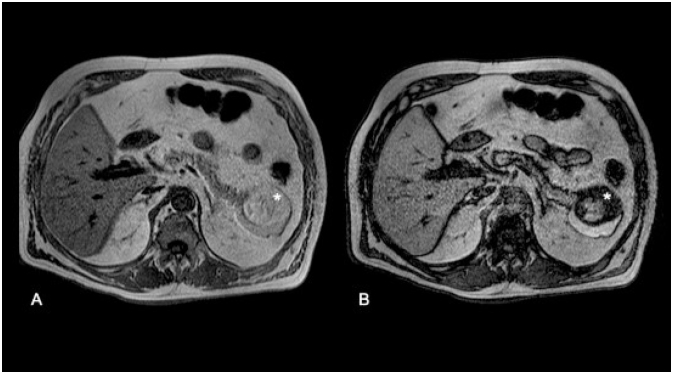
Axial T1-weighted images. (A) In-phase. (B). Out-of-phase. Signal intensity dropout in In-phase/Out-of-phase sequences shows microscopic fat component within the tumoral cystic cavity (*).

**Figure 4:**
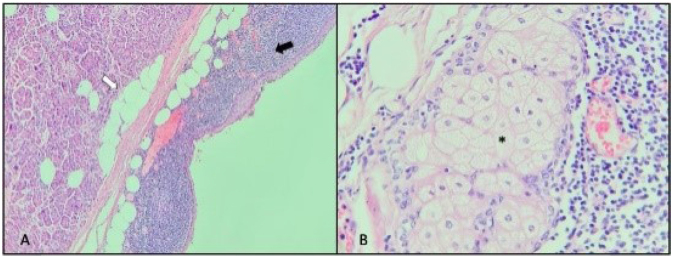
Microscopic analysis. (A) The diffuse cystic area was composed of mature adipose tissue (white arrow) lined by squamous cells from the wall (black arrow) (100´; H&E stain). (B) In the central zone of tumor, well-differenced adnexal tissue (*) is surrounded by inflammatory pancreatic tissue (400´; H&E stain).

A 72-year-old man with medical history of type 2 diabetes mellitus presented to our institution for the evaluation of an incidentally detected pancreatic mass in the absence of abdominal complaints, jaundice, or weight loss history. On physical examination, the abdomen was tender and no abdominal masses were palpable. Initially, laboratory test showed normal values for bilirubin, CA-19-9, and liver function tests. Gadolinium-enhanced abdominal magnetic resonance imaging (MRI) depicted an exophytic and well-circumscribed multinodular mass arising from pancreas tail (Figure 1), with asymmetric parietal enhancement and a heterogeneous and hyperintense signal in T1- and T2-weighted images, in the absence of bile and pancreatic ductal alteration, as well as malignant suspected lesions (Figures 2 and 3). Considering the caudal localization and solid-cystic features of tumor, we planned to perform a laparoscopic distal pancreatectomy (LDP) and splenectomy without lymph node dissection in sequence. First, we performed dissection of greater omentum with gastric retraction in order to obtain good exposure and subsequent liberation of anteroinferior edge using a multistep clockwise technique, by finding a complex solid-cystic tumor arising from the uncinated process, which was completely resected and sent for histopathological analysis. Macroscopically, the pathological evaluation presented a unilocular cavity of 7.5´5´2 cm in size, nearly to the uncinated process, which contains a solid heterogeneous content surrounded by inflammatory pancreatic tissue. Histopathological analysis reveals a complex and well-defined solid-cystic mass encapsulated on a wall lined by a squamous epithelial. Internally, the cyst contained abundant epithelial cells admixed with mature adipose tissue, keratinous debris, and anucleated squamous cells in addition to highly differentiated adnexal cells and multiple sebaceous focal areas with lymphocytic infiltration, consistent with a diagnosis of mature cystic teratoma associated with extensive acinar-ductal metaplasia and chronic pancreatitis with multiple epithelial fibrosis zones. There was no evidence of malignancy (Figure 4). The postoperative period was uneventful, and the patient was discharged on day 4. At 12-month follow-up, the patient was asymptomatic, without any complain or evidence of recurrence disease. Written informed consent was obtained from the patient for publication of this case and any accompanying images (CEC-01 Servicio de Salud del Maule Scientific Ethics Committee 61606900-4).

## DISCUSSION

Teratomas are an uncommon type of germ cell-derived neoplasm, which have highly heterogeneous biological behavior according to its histological origin^9,13^. This type of neoplasm is commonly found in the ovary, testis, retroperitoneum, and/or mediastinum, but they may arise anywhere along the path of germ cell migration during embryogenesis, usually along the midline of the body^6^. Strikingly, the pancreas is considered the least common site of presentation, with less than 60 clinical cases reported in the most recent literature review^7,10,11^. This type of tumor is usually diagnosed in younger patients, with a mean age of 37 years at diagnosis, ranging widely from 4 months to 74 years. Some clinical series provide evidence that male patients show a slight predominance than female patients^7,9,13^. Among all age groups, most patients present clinically asymptomatic or with nonspecific systemic or gastrointestinal complaints, including fatigue, nausea, vomiting, weight loss history, and abdominal or back pain^14^. At physical examination, palpable abdominal mass and/or abdominal tenderness are the most common findings. During laboratory test evaluation, results are usually normal, except for cases where there is biliary or pancreatic ductal communication or obstruction^4,8^. Although cases have been identified throughout the pancreas, pancreatic cystic tumors are more commonly described in the pancreatic body and head area^1,5^. In addition, as a slow-growing neoplasm, the pancreatic cystic teratomas are usually large at its clinical presentation, with a mean dimension of approximately 7–8 cm in long axis, ranging from 2.2 to 22 cm^5,10^. At present, the preoperative diagnosis of this type of tumors remains still difficult because of their extremely rare nature, especially when it arises from uncommonly sites, such pancreas tail^5^. However, the use of combined clinical imaging methods might allow differentiation of some pancreatic cystic lesions with increased risk of occult malignancy^2,13^. Despite the fact that the malignant degeneration of pancreatic mature cystic teratoma has never been reported, complete surgical resection procedures such as duodenopancreatectomy and distal pancreatectomy remain the current standard of care and can be regarded as save procedures^13,15^. Although the minimally invasive technique might increase the surgical time compared with open distal pancreatectomy (ODP), LDP is known as a safe procedure that not only significantly reduces the intraoperative blood loss but also improves the outcomes in postoperative feeding advance^15^. In this context, compelling body of evidence supports LDP as the first-line treatment for this type of cystic tumors in the left side of the pancreas^3,12^.
